# Blood Transfusion Predicts Prolonged Mechanical Ventilation in Acute Stanford Type A Aortic Dissection Undergoing Total Aortic Arch Replacement

**DOI:** 10.3389/fcvm.2022.832396

**Published:** 2022-04-15

**Authors:** Qiang Xie, Chengnan Li, Yongliang Zhong, Congcong Luo, Rutao Guo, Yongmin Liu, Jun Zheng, Yipeng Ge, Lizhong Sun, Junming Zhu

**Affiliations:** Department of Cardiovascular Surgery, Beijing Aortic Disease Center, Beijing Anzhen Hospital, Capital Medical University, Beijing, China

**Keywords:** blood transfusion, prolonged mechanical ventilation (PMV), acute Stanford type A aortic dissection (ATAAD), total aortic arch replacement (TAR), risk factor

## Abstract

**Background:**

This research aimed to evaluate the impacts of transfusing packed red blood cells (pRBCs), fresh frozen plasma (FFP), or platelet concentrate (PC) on postoperative mechanical ventilation time (MVT) in patients with acute Stanford type A aortic dissection (ATAAD) undergoing after total arch replacement (TAR).

**Methods:**

The clinical data of 384 patients with ATAAD after TAR were retrospectively collected from December 2015 to October 2017 to verify whether pRBCs, FFP, or PC transfusion volumes were associated with postoperative MVT. The logistic regression was used to assess whether blood products were risk factors for prolonged mechanical ventilation (PMV) in all three endpoints (PMV ≥24 h, ≥48 h, and ≥72 h).

**Results:**

The mean age of 384 patients was 47.6 ± 10.689 years, and 301 (78.39%) patients were men. Median MVT was 29.5 (4–574) h (h), and 213 (55.47%), 136 (35.42%), and 96 (25.00%) patients had PMV ≥24 h, ≥48 h, and ≥72 h, respectively. A total of 36 (9.38%) patients did not have any blood product transfusion, the number of patients with transfusion of pRBCs, FFP, and PC were 334 (86.98%), 286 (74.48%), and 189 (49.22%), respectively. According to the multivariate logistic regression of three PMV time-endpoints, age was a risk factor [PMV ≥ 24 h odds ratio (*OR*_PMV≥24_) = 1.045, *p* = 0.005; *OR*_PMV≥48_ = 1.060, *p* = 0.002; *OR*_PMV≥72_ = 1.051, *p* = 0.011]. pRBC transfusion (*OR*_PMV≥24_ = 1.156, *p* = 0.001; *OR*_PMV≥48_ = 1.156, *p* < 0.001; *OR*_PMV≥72_ = 1.135, *p* ≤ 0.001) and PC transfusion (*OR*_PMV≥24_ = 1.366, *p* = 0.029; *OR*_PMV≥48_ = 1.226, *p* = 0.030; *OR*_PMV≥72_ = 1.229, *p* = 0.011) were independent risk factors for PMV. FFP had no noticeable effect on PMV [*OR*_PMV≥48_ = 0.999, 95% confidence interval (*CI*) 0.998–1.000, *p* = 0.039; *OR*_PMV≥72_ = 0.999, 95% *CI*: 0.998–1.000, *p* = 0.025].

**Conclusions:**

In patients with ATAAD after TAR, the incidence of PMV was very high. Blood products transfusion was closely related to postoperative mechanical ventilation time. pRBC and PC transfusions and age increased the incidence of PMV at all three endpoints.

## Introduction

Acute Stanford type A aorta dissection (ATAAD) is a fatal cardiovascular disease. Due to the widespread lesion, the mortality with immediate surgical intervention is still as high as 30% and drug treatment is as high as 58% (1, 2). Total arch replacement (TAR) is a common approach for ATAAD. As a complex, traumatic operation, there are complications of multiple organs, such as the lungs and the kidney, after the operation (3, 4). Mechanical ventilation (MV) helps patients after TAR to improve oxygen saturation and to prevent lung atelectasis (5). However, many studies showed that the physiological state, drug use, and postoperative complications lead to a high probability of prolonged mechanical ventilation (PMV), which increases infection and mortality (6, 7). Almost all patients with ATAAD require different blood products to improve circulation and correct anemia and hypoproteinemia intraoperatively and postoperatively (8). It is still unclear whether changes in the internal environment brought by different blood products will affect mechanical ventilation time (MVT). This research attempted to explore whether blood product transfusion is a predictor of PMV in ATAAD patients undergoing TAR.

## Materials and Methods

### Patients

A total of 384 consecutive patients (47.6 ± 10.689 years) with ATAAD undergoing TAR were enrolled at Beijing Anzhen Hospital of Capital Medical University from December 2015 to October 2017. The inclusion criteria were as follows: patients who had been diagnosed as Stanford type A aortic dissection by aortic enhanced CT and who underwent surgical treatment; those with the onset of disease at ≤14 days; and those aged ≥18 years. Patients with traumatic aortic dissection, those with aortic dissection during pregnancy, those with the onset of disease at more than 14 days, and those aged <18 were excluded. All the clinical data were retrospectively recorded from the medical records; 33 cases had been eliminated with insufficient data. This retrospective study was approved by the Medical Ethical Committee of the Beijing Anzhen Hospital of Capital Medical University (2020100X) and waived the need for individual patient consent.

### Mechanical Ventilation Management

The Society of Thoracic Surgeons and most studies define PMV as mechanical ventilation for 24 h (h) or more (9, 10). In our research, by combining preoperative and postoperative clinical parameters in patients with ATAAD undergoing TAR, we assessed different periods of MVT (≥24 h, ≥48 h, and ≥72 h) for outcomes. We adjusted the ventilator parameters appropriately to the patient's respiratory oxygenation by maintaining SaO_2_ > 90% or PaO_2_ > 60 mmHg (11, 12). The extubation decision was the consulting anesthetist's independent discretion, usually after the spontaneous breathing or under a low-level pressure support trial. When the tracheal tube was removed, hemodynamic parameters were stable without tachypnea, anxiety, sweating, or descent.

### Blood Products and Indications

The respective totality of packed red blood cells (pRBCs), fresh frozen plasma (FFP), or platelet concentrate (PC) that patients transfused intraoperatively and postoperatively were recorded. The total number of pRBCs units (U) did not include autologous blood stored intraoperatively and postoperatively. Indications for pRBCs transfusion included the hemoglobin level of ≤9.0 g/dl on the surgery day or ≤7.0 g/dl postoperatively. The PC transfusion indications included life-threatening active bleeding, the platelet count ≤ 50,000/ml, or the platelet count <100,000/ml with bleeding. The FFP was transfused when the prothrombin time was >1.5 times the upper limit of normal with a prolonged cardiopulmonary bypass (CPB) time or clinical signs of bleeding (13–15).

### Total Arch Replacement (TAR)

All procedures were median sternotomy, and total CPB with selective antegrade cerebral perfusion (SCP) was performed in the right axillary artery and the right atrium. The arterial hemoperfusion consisted of right axillary artery perfusion and one branch of a four-branch aortic graft. The ascending aorta was clamped during cooling, and a cold-blood cardioplegic solution was antegrade infused into the coronary ostia by a longitudinal incision in the proximal ascending aorta. An anastomosis of the four-branch aortic graft to the aortic root was finished in the cooling phase; the circulatory anastomosis was arrested after the nasopharyngeal temperature reached 25°C. After the brachiocephalic arteries were clamped, unilateral SCP was started through the right axillary artery. The anastomosis of the stent graft in the aortic arch to the four-branch graft distal end was completed, and the four-branch graft was clamped. The lower body got blood perfusion *via* the perfusion limb of the four-branch graft. SCP was discontinued after anastomosis of the left common carotid artery, the left subclavian artery, and the innominate artery. Then, CPB flow was gradually resumed normal and began to warm.

### Statistical Analysis

Descriptive statistics were expressed as means ± standard deviations or median (interquartile range) for continuous variables and percentages and frequencies for categorical variables. Independent-samples *t*-test and non-parametric tests were used to compare continuous variables. The categorical variables were compared by the χ^2^ analysis or Fisher's exact correction. The *p*-value of <0.05 was considered significant. Then, a stepwise multivariable logistic regression analysis was used to determine factors independently associated with PMV. Statistical Package for the Social Sciences Version 26.0 (IBM Corp, Armonk, NY, USA) was used to analyze data. Prism 9 (GraphPad Software, San Diego, California, USA) was used for drawing figures.

## Results

### Patients Baseline Characteristics

The age (mean ± standard deviation) of all 384 patients was 47.64 ± 10.69 years, 301 (78.39%) patients were men, and time (median, interquartile range) from related symptom onset to TAR was 24 (13.00–48.00) h. The median CPB time was 204.50 (180.00–234.00) min (min), and 18 (4.70%) patients had the median CPB time of over 300 min A total of 27 (7.00%) patients received recombinant factor VIIa (rFVIIa) infusion in-hospital, and the median volume of the total intraoperative and postoperative of pRBCs, FFP, and PC was 8.00 (4.00–14.00) U, 400.00 (0–800.00) ml, and 0 (0–2.00) *U*, respectively. Median MVT was 29.50 (14.00–71.75) h, the number of cases of PMV ≥24 h, ≥48 h, and ≥72 h was 213 (55.47%), 136 (35.42%), and 96 (25.00%), respectively. More perioperative baseline data and main complications characteristics were shown in Table 1.

**Table 1:** Baseline characteristics and perioperative data of the patients (*n* = 384).

**Variables**	**Mean/Median/*n* (%)**		**Mean/Median/*n* (%)**
Pre-operative		Intra-/post-operative	
Age (years)	47.64 ± 10.69	Bypass (*n*)	10.00 (2.60%)
Male sex (*n*)	301.00 (77.80%)	CPB time (mins)	204.50 (180.00-234.00)
BMI (kg/m^2^)	26.00 (24.00-29.00)	CPB ≥300 mins (*n*)	18.00 (4.70%)
Hypertension (*n*)	288.00 (75.00%)	Cross-clamp time (mins)	112.50 (94.00-136.75)
IDDM (*n*)	19.00 (4.90%)	CPB arrest time (mins)	21.00 (17.00-27.00)
Surgical period from symptom onset (hours)	24.00 (13.00-48.00)	Intra-operative blood loss (mL)	1500.00 (1200.00-2000.00)
Renal artery involvement (*n*)	307.00 (79.90%)	Post-operative suction drainage (mL/12h)	500.00 (300.00-867.50)
LVEF (%)	60.00 (58.00-65.00)	rFVIIa (*n*)	27.00 (7.00%)
LVEDD (mm)	50.00 (46.00-54.00)	pRBCs (units)	8.00 (4.00-14.00)
AST (U/L)	23.00 (17.00-33.00)	FFP (mL)	400.00 (0-800.00)
ALT (U/L)	24.00 (19.00-34.00)	PC (units)	0 (0-2.00)
CR (μmol/L)	79.90 (66.95-98.23)	MVT (hours)	29.50 (14.00-71.75)
CC (mL/min)	105.18 (79.97-129.27)	Main complications	
CC ≤85 mL/min (*n*)	107.00 (27.90%)	AKI (*n*)	203.00 (52.90%)
DDIMER (ng/mL)	2144.50 (962.5.00-3363.50)	KIDGO3 (*n*)	77.00 (20.10%)
PLT (*10^9^/L)	173.00 (137.25-206.75)	CRRT (*n*)	61.00 (15.90%)
WBC (*10^9^/L)	11.47 (9.35-14.40)	Secondary thoracotomy (*n*)	38.00 (9.90%)
HgB (g/L)	138.00 (127.00-148.00)	Stroke (*n*)	40.00 (10.40%)
		Paraplegia and paraparesis (*n*)	25.00 (6.50%)
		Mortality (*n*)	29.00 (7.60%)

*BMI, body mass index; IDDM, insulin-dependent diabetes mellitus; LVEF, left ventricular ejection fraction; LVEDD, left ventricular end-diastolic dimension; AST, aspartate aminotransferase; ALT, alanine aminotransferase; CR, creatinine CC, creatinine clearance; PLT, platelet count; WBC, white blood cells; HgB, hemoglobin; CPB, cardiopulmonary bypass; rFVIIa: recombinant factor VIIa; pRBCs, packed red blood cells; FFP, fresh frozen plasma; PC, platelet concentrate; MVT, mechanical ventilation time; AKI, acute kidney injury; KDIGO, Kidney Disease: Improving Global Outcomes; CRRT, continuous renal replacement therapy*.

*Data were present as n (%), mean ± standard deviation, or median (interquartile range) according to variable category*.

### Blood Products Transfusion and MVT

**Figure 1:**
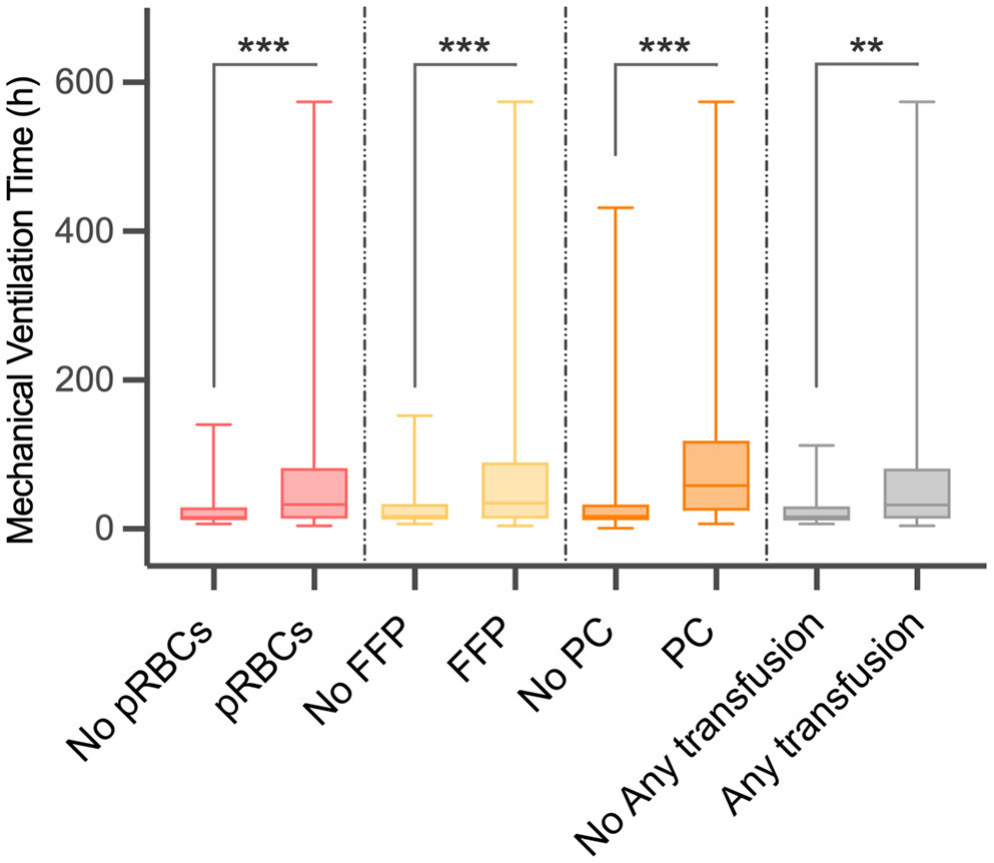
Difference in mechanical ventilation time (MVT) between different blood product transfusion and no transfusion. MVT, mechanical ventilation time; pRBCs, packed red blood cells; FFP, fresh frozen plasma; PC, platelet concentrate. ^**^p < 0.01, ^***^p < 0.001.

**Figure 2:**
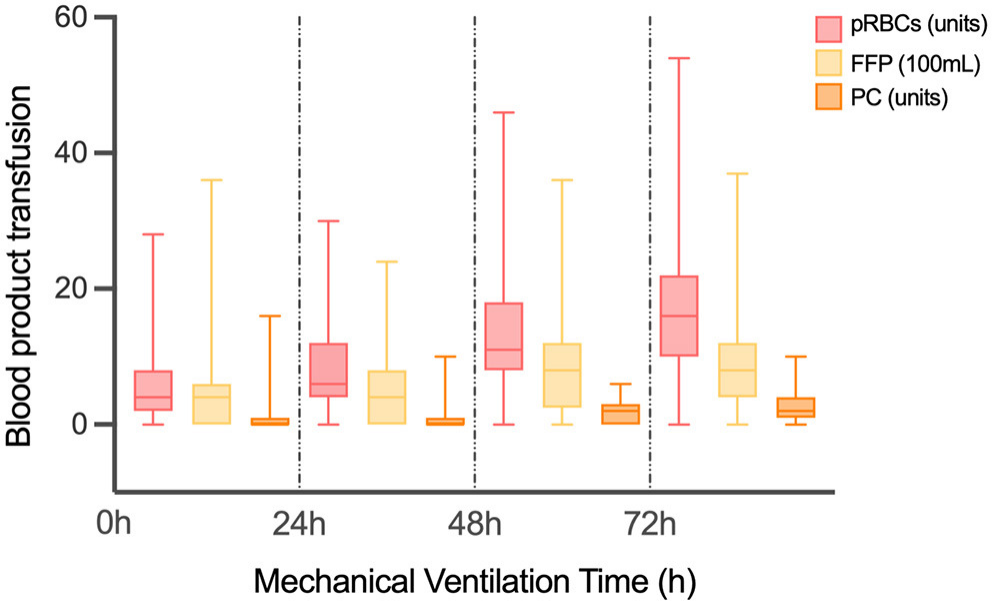
The volume of different transfusion in patients with different MVT. MVT, mechanical ventilation time; pRBCs, packed red blood cells; FFP, fresh frozen plasma; PC, platelet concentrate.

As shown in Table 2 and Figure 1, there were 50 (13.02%) patients without any pRBCs infusion, 97 (25.26%) patients without FFP, and 194 (50.52%) cases without PC. Only 36 (9.38%) cases did not have any transfusion, and the MVT difference in transfusion (16.50, 11.25–30.50 h) or without transfusion (32.00, 14.00–81.00 h) was significant (*p* = 0.001). In different periods of PMV (≥24 h, ≥48 h, and ≥72 h), the respective transfusion volume of pRBCs, FFP, or PC was statistically different (Table 3; Figure 2).

**Table 2:** Differences in MVT after different blood products transfusion.

**Variables**	**Mechanical ventilation time (hours)**	***P*-value**
pRBCs		**<0.001**
No (*n* = 50, 13.02%)	16.00 (11.75–29.25)	
Yes (*n* = 334, 86.98%)	33.00 (14.00–82.00)	
FFP		**<0.001**
No (*n* = 97, 25.26%)	17.00 (12.50–32.50)	
Yes (*n* = 287, 74.74%)	35.00 (14.00–90.00)	
PC		**<0.001**
No (*n* = 194, 50.52%)	17.00 (12.00–33.25)	
Yes (*n* = 190, 49.48%)	58.00 (24.75–120.25)	
Any transfusion		**0.001**
No (*n* = 36, 9.38%)	16.50 (11.25–30.50)	
Yes (*n* = 348, 90.62%)	32.00 (14.00–81.00)	

*MVT, mechanical ventilation time; pRBCs, packed red blood cells; FFP, fresh frozen plasma; PC, platelet concentrate*.

*Data were present as n (%), or median (interquartile range) according to variable category. Differences were considered statistically significant for P-values of < 0.05, which are shown in bold*.

**Table 3:** The differences in blood products transfusion volume in different MVT.

**Variables**	**Mechanical ventilation time**				**H-value**	***P* value**
	**0 h < MVT <24 h**	**24 h≤MVT <48 h**	**48 h≤MVT <72 h**	**72 h≤MVT**		
	**(171, 44.53%)**	**(77, 20.05%)**	**(40, 10.42%)**	**(96, 25.00%)**		
pRBCs (units)	4.00 (2.00–8.00)	6.00 (4.00–12.00)	11.00 (8.00–18.00)	16.00 (10.00–22.00)	113.89	**<0.001**
FFP (mL)	400.00 (0–600.00)	400.00 (0–800.00)	700.00 (107.00–1,200.00)	800.00 (400.00–1,200.00)	48.11	**<0.001**
PC (units)	0 (0–1.00)	0 (0–1.00)	2.00 (0–3.00)	2.00 (1.00–4.00)	115.88	**<0.001**

*MVT, mechanical ventilation time; pRBCs, packed red blood cells; FFP, fresh frozen plasma; PC, platelet concentrate*.

*Data were present as median (interquartile range) according to variable category. Differences were considered to be statistically significant for P-values of < 0.05 which are shown in bold*.

### PMV and Clinical Characteristics

In Table 4, the relation of different PMV (≥24 h, ≥48 h, and ≥72 h) and perioperative factors were assessed. Several indicators were noteworthy, such as the surgical period from disease onset showed an effect on PMV ≥ 48 h (*p* = 0.003), but not on PMV ≥24 h or ≥72 h. PMV ≥24 h and ≥48 h were affected (*p* = 0.048, *p* = 0.013, respectively) by renal artery involved dissection, but not PMV ≥72 h. Left ventricular end-diastolic dimension showed a statistical significance (*p* = 0.032) only in PMV ≥72 h. The platelet (PLT) count was different (*p* = 0.001, *p* = 0.001, respectively) both in PMV ≥24 h and ≥48 h. When the CPB was ≥300 min, the difference (*p* = 0.020) was only shown in PMV ≥48 h. The transfusion of rFVIIa, pRBCs, FFP, and PC, respectively, showed a statistical difference on three PMV.

**Table 4:** Baseline characteristics and perioperative data in three PMV time-endpoints.

**Variables**	**PMV** **≥24 h**		***P*-value**	**PMV** **≥48 h**		***P*-value**	**PMV** **≥72 h**		***P*-value**
	**No (*n* = 171)**	**Yes (*n* = 213)**		**No (*n* = 248)**	**Yes (*n* = 136)**		**No (*n*=288)**	**Yes (*n*=96)**	
**Pre-operative**									
Age (years)	44.87 ± 9.444	49.86 ± 11.123	**<0.001**	45.58 ± 10.003	51.40 ± 10.908	**<0.001**	46.28 ± 10.435	51.72 ± 10.446	**<0.001**
Male sex (*n*)	141 (82.5%)	160 (75.1%)	0.082	197 (79.4%)	104 (76.5%)	0.500	232 (80.6%)	69 (71.9%)	0.074
BMI (kg/m^2^)	26.00 (23.00–28.00)	26.00 (24.00–29.00)	0.575	26.00 (24.00–28.75)	26.00 (23.00–29.00)	0.833	26.00 (24.00–29.00)	26.00 (23.00–28.75)	0.862
Hypertension (*n*)	126 (73.7%)	162 (76.1%)	0.594	183 (73.8%)	105 (77.2%)	0.460	212 (73.6%)	76 (79.2%)	0.276
IDDM (*n*)	9 (5.3%)	10 (4.7%)	0.799	11 (4.4%)	8 (5.9%)	0.532	13 (4.5%)	6 (6.3%)	0.684
Surgical period from symptom onset (hours)	24.00 (13.00–72.00)	24.00 (14.00–48.00)	0.097	24.00 (15.00–72.00)	24.00 (12.00–36.00)	**0.003**	24.00 (14.25–69.50)	24.00 (12.00–24.00)	0.862
Renal artery involvement (*n*)	129 (75.4%)	178 (83.6%)	**0.048**	189 (76.2%)	118 (86.8%)	**0.013**	224 (77.8%)	83 (86.5%)	0.066
LVEF (%)	60.00 (59.00–65.00)	60.00 (58.00–65.00)	0.249	60.00 (58.00–65.00)	60.00 (57.25–65.00)	0.203	60.00 (58.00–65.00)	60.00 (57.00–65.00)	0.121
LVEDD (mm)	50.00 (47.00–54.00)	49.00 (45.50–54.00)	0.135	50.00 (46.00–54.00)	49.00 (44.25–54.00)	0.099	50.00 (46.00–54.00)	48.00 (44.00–53.75)	**0.032**
AST (unit/L)	22.00 (16.00–29.00)	25.00 (17.00–38.00)	**0.007**	22.00 (16.00–30.00)	26.00 (19.00–39.75)	**0.001**	22.00 (16.00–30.75)	27.50 (19.00–43.75)	**0.001**
ALT (unit/L)	22.00 (17.00–31.00)	25.00 (20.00–35.00)	**0.021**	22.00 (17.00–31.00)	27.00 (21.00–39.00)	**<0.001**	23.00 (18.00–31.00)	27.00 (22.00–40.75)	**<0.001**
CR (μmol/L)	75.60 (63.40–88.10)	83.40 (69.20–109.85)	**<0.001**	78.40 (63.33–93.78)	82.90 (69.70–108.98)	**0.002**	78.95 (65.20–95.330)	84.95 (70.20–115.03)	**0.007**
CC (ml/min)	113.39 (95.00–134.24)	96.21 (71.48–119.60)	**<0.001**	111.50 (90.04–133.47)	91.73 (67.94–115.13)	**<0.001**	108.71 (86.22–133.27)	95.03 (64.29–112.15)	**<0.001**
CC ≤85 mL/min (*n*)	28 (16.4%)	79 (37.1%)	**<0.001**	52 (21.0%)	55 (40.4%)	**<0.001**	68 (23.6%)	39 (40.6%)	**0.001**
DDIMER (ng/ml)	1,261.00 (728.00–2,716.00)	2,574.00 (1,167.50–8,301.50)	**<0.001**	1,768.50 (813.25–2,771.75)	2,792.50 (1,343.50–69,392.25)	**<0.001**	1,887.00 (854.00–2,887.50)	2,938.00 (1,475.75–9,571.50)	**<0.001**
PLT (*10^9^/L)	180.00 (150.00–215.00)	164.00 (131.00–196.50)	**0.001**	178.00 (145.00–211.00)	158.00 (129.00–189.75)	**0.001**	175.00 (142.00–207.00)	163.00 (132.25–199.50)	0.115
WBC (*10^9^/L)	10.95 (8.53–13.79)	12.00 (10.02–14.98)	**<0.001**	11.12 (8.60–14.11)	12.00 (10.30–14.77)	**0.003**	11.30 (8.93–14.06)	12.27 (10.33–15.45)	**0.001**
HgB (g/L)	141.00 (129.00–149.00)	137.00 (125.00–147.50)	0.065	139.50 (127.25–149.00)	137.00 (126.25–147.00)	0.249	139.00 (127.25–149.00)	137.50 (124.50–146.00)	0.165
**Intra-/post-operative**									
Bypass (*n*)	3 (1.8%)	7 (3.3%)	0.349	8 (3.2%)	2 (1.5%)	0.302	8 (2.8%)	2 (2.1%)	0.712
CPB time (mins)	197.00 (174.00–222.00)	209.00 (186.00–245.00)	**<0.001**	198.00 (105.00–387.00)	214.50 (131.00–450.00)	**<0.001**	201.00 (177.00–229.00)	214.50 (189.00–248.50)	**0.001**
CPB≥300 mins (*n*)	4 (2.3%)	14 (6.6%)	0.051	7 (2.8%)	11 (8.1%)	**0.020**	10 (3.5%)	8 (8.3%)	0.094
Cross-clamp time (mins)	109.00 (90.00–129.00)	115.00 (96.00–145.50)	**0.002**	109.00 (90.00–131.00)	118.50 (100.00–144.00)	**<0.001**	110.00 (92.00–132.00)	118.00 (98.00–143.75)	**0.021**
CPB arrest time (mins)	20.00 (17.00–27.00)	21.00 (17.50–27.00)	0.270	21.00 (17.00–27.00)	21.00 (18.00–26.75)	0.521	21.00 (17.00–27.00)	21.00 (18.00–26.75)	0.839
Intra-operative blood loss (mL)	1,300.00 (1,000.00–1,600.00)	1,500.00 (1,200.00–2,000.00)	**<0.001**	1,400.00 (1,000.00–1,800.00)	1,600.00 (1,200.00–2,000.00)	**<0.001**	1,500.00 (1,000.00–1,800.00)	1,600.00 (1,200.00–2,000.00)	**<0.001**
Post-operative suction drainage (mL/12h)	440.00 (260.00–700.00)	610.00 (350.00–1,000.00)	**<0.001**	450.00 (262.50–700.00)	675.00 (427.50–1,200.00)	**<0.001**	462.50 (270.00–747.500)	675.00 (450.00–1,117.50)	**<0.001**
rFVIIa (*n*)	5 (2.9%)	22 (10.3%)	**0.005**	10 (4.0%)	17 (12.5%)	**0.002**	14 (4.9%)	13 (13.5%)	**0.004**
pRBCs (units)	4.00 (2.00–8.00)	12.00 (6.00–20.00)	**<0.001**	4.50 (2.00–8.00)	14.00 (8.00–22.00)	**<0.001**	6.00 (4.00–10.00)	16.00 (10.00–22.00)	**<0.001**
FFP (mL)	400.00 (0–600.00)	600.00 (400.00–1,000.00)	**<0.001**	400.00 (0–600.00)	800.00 (400.00–1,200.00)	**<0.001**	400.00 (0–800.00)	800.00 (400.00–1,200.00)	**<0.001**
PC (units)	0 (0–1.00)	1.00 (0–3.00)	**<0.001**	0 (0–1.00)	2.00 (1.00–3.75)	**<0.001**	0 (0–1.00)	2.00 (1.00–4.00)	**<0.001**

*PMV, prolonged mechanical ventilation; BMI, body mass index; IDDM, insulin-dependent diabetes mellitus; LVEF, left ventricular ejection fraction; LVEDD, left ventricular end-diastolic dimension; AST, aspartate aminotransferase; ALT, alanine aminotransferase; CR, creatinine CC, creatinine clearance; PLT, platelet count; WBC, white blood cells; HgB, hemoglobin; CPB, cardiopulmonary bypass; rFVIIa: recombinant factor VIIa; pRBCs, packed red blood cells; FFP, fresh frozen plasma; PC, platelet concentrate; MVT, mechanical ventilation time; AKI, acute kidney injury; KDIGO, Kidney Disease: Improving Global Outcomes; CRRT, continuous renal replacement therapy. Data were present as n (%), mean ± standard deviation, or median (interquartile range) according to variable category. Differences were considered statistically significant for P-values of < 0.05, which are shown in bold*.

### Risk Factors of PMV

Multivariate logistic regression with three different PMV (≥24 h, ≥48 h, and ≥72 h) endpoints showed that age was a risk factor [PMV ≥ 24 h, odds ratio (*OR*_PMV≥24_) = 1.045, *p* = 0.005; *OR*_PMV≥48_ = 1.060, *p* = 0.002; *OR*_PMV≥72_ = 1.051, *p* = 0.011]. pRBC (*OR*_PMV≥24_ = 1.156, *p* = 0.001; *OR*_PMV≥48_ = 1.156, *p* < 0.001; *OR*_PMV≥72_ = 1.135, *p* ≤0.001) and PC (*OR*_PMV≥24_ = 1.366, *p* = 0.029; *OR*_PMV≥48_ = 1.226, *p* = 0.030; *OR*_PMV≥72_ = 1.229, *p* = 0.011) transfusions were independent risk factors for PMV (Table 5). However, FFP had no significant effect on PMV ≥24 h [*OR* = 0.999, 95% confidence interval (*CI*): 0.999–1.000, *p* = 0.086], on PMV ≥48 h (*OR* = 0.999, 95% *CI*: 0.998–1.000, *p* = 0.039), and PMV ≥72 h (*OR* = 0.999, 95% *CI*: 0.998–1.000, *p* = 0.025). Besides, while considering PMV ≥24 h as the endpoint, AST (*OR* = 1.022, *p* = 0.022), ALT (*OR* = 0.986, *p* = 0.039), and WBC (*OR* = 1.123, *p* = 0.002) were preoperative risk factors. The CPB time (*OR* = 1.013, *p* = 0.042) was the predictive factor of PMV ≥48 h. For PMV ≥72 h, WBC (*OR* = 1.116, *p* = 0.012) had the predictive effect.

**Table 5:** Multivariate analysis for three PMV time-endpoints.

**Risk factors**	**OR**	**95% CI**	***P*-value**
PMV ≥24 h			
Age	1.045	1.014–1.077	**0.005**
AST	1.022	1.003–1.041	**0.022**
ALT	0.986	0.972–0.999	**0.039**
WBC	1.123	1.042–1.210	**0.002**
pRBCs	1.156	1.082–1.234	**<0.001**
PC	1.367	1.034–1.808	**0.028**
PMV ≥48 h			
Age	1.060	1.023–1.099	**0.002**
CPB	1.013	1.000–1.025	**0.042**
pRBCs	1.156	1.092–1.224	**<0.001**
FFP	0.999	0.999–1.000	**0.043**
PC	1.229	1.022–1.478	**0.028**
PMV ≥72 h			
Age	1.051	1.012–1.092	**0.010**
WBC	1.116	1.025–1.215	**0.012**
pRBCs	1.135	1.078–1.195	**<0.001**
FFP	0.999	0.999–1.000	**0.030**
PC	1.231	1.051–1.442	**0.010**

*PMV, prolonged mechanical ventilation; OR, odds ratio; CI, confidence interval; AST, aspartate aminotransferase; ALT, alanine aminotransferase; WBC, white blood cells; pRBCs, packed red blood cells; PC, platelet concentrate; CPB, cardiopulmonary bypass; FFP, fresh frozen plasma*.

*Differences were considered statistically significant for P-values of < 0.05, which are shown in bold*.

### Blood Transfusion and Other Adverse Events

**Figure 3:**
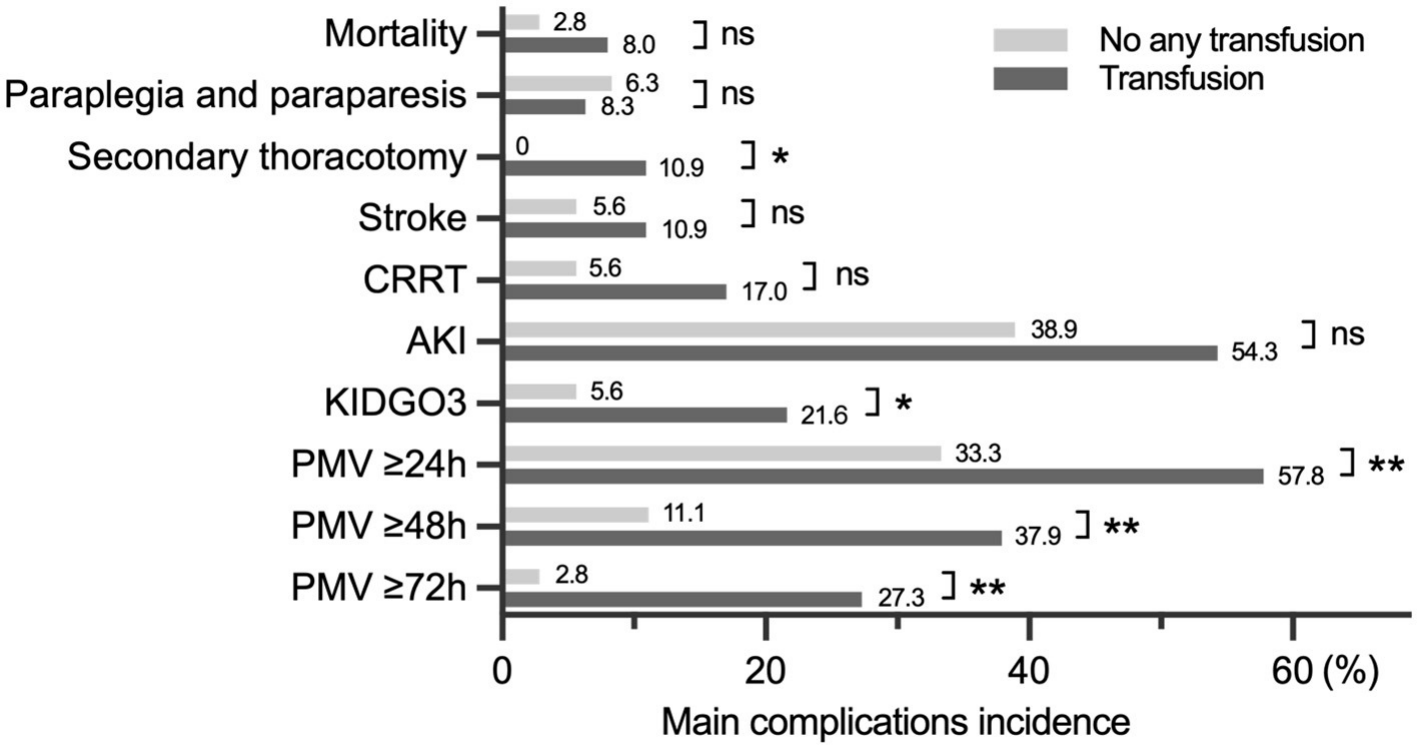
The difference in the incidence of complications between transfusion and no transfusion. CRRT, continuous renal replacement therapy; AKI, acute kidney injury; KDIGO, kidney disease improving global outcomes; PMV, prolonged mechanical ventilation. *p < 0.05, **p < 0.01.

In addition to pRBCs transfusion volume between the paraplegic and non-paraplegic, the difference in blood transfusion volume between with and without different complications was statistically significant (Supplementary Table 1). The difference in the incidence of adverse events such as PMV) between patients with and without blood transfusion was evaluated, with secondary thoracotomy, kidney disease: improving global outcomes criteria stage 3 (KIDGO3), PMV showing statistical significance (Figure 3).

## Discussion

Mechanical ventilation can help to recover patients' cardiopulmonary function and circulation quickly after a long, traumatic, and complex operation. However, timely extubation is necessary to avoid mechanical ventilation complications such as infection, trachea damage, and ventilator dependence (6, 7). This study found that MVT exceeded 24 h in 213 (55.47%) cases, 48 h in 136 (35.42%) cases, and 72 h in 96 (25.00%) cases, which is the first time that PMV incidence of different endpoints in ATAAD after TAR were reported. Such PMV incidence was significantly higher than other cardiovascular operations, such as coronary artery bypass grafting and the tetralogy of Fallot (16–18). More studies focused on finding PMV predictors after cardiovascular surgery in blood indicators, CPB process, and cardiac function for patients (16, 19). This study is the first attempt to identify risk factors from three different PMV endpoints for MVT after TAR for severe aortic dissection.

During intraoperative and postoperative aortic surgery, different blood products often need to be infused to improve circulation and prevent multiple organ injuries. Before this, only two studies attempted to explain the relationship between perioperative blood transfusions and PMV during cardiovascular surgery. In one study, PMV ≥ 48 h was correlated with intraoperative blood transfusion in an open-heart surgery (20). However, it neither did provide detailed data on blood products type and transfusion volume nor did it record the transfusion of patients in ICU. Another study showed that the blood products transfusion ≥2,000 ml in ATAAD repair was correlated with PMV ≥48 h, but had limitations such as incomplete blood products data and too few patients involved in comparison (18). These reference values were not evident.

In our study of 384 cases, the MVT was significantly different between the groups with or without transfusion of different blood products (such as pRBCs, FFP, PC, or anyone), and the respective transfusion volume of pRBCs, FFP, or PC in four different MVT groups had statistical significance. Moreover, after summarizing three multivariate logistics regressions of different PMV endpoints, it was found that intraoperative and postoperative pRBC and PC transfusions are effective predictors for PMV (≥24 h, ≥48 h, and ≥72 h). For lack of targeted research, the mechanism by which pRBC and PC transfusions affect PMV remains unclear. According to the existing studies, we speculated that it might be related to the decreased deformability and oxygen transfer of the allogeneic stored red cells, and the hemolysis of the long-stored red cells will lead to electrolyte disorder (the free hemoglobin, iron, and K^+^ levels, especially) and coagulation dysfunction (21–23). PC contained many different pyrogenic and inflammatory factors that can cause severe fever or even infection in patients with compromised immunity after cardiovascular surgery (24–26). These are all possible causes of delay extubation of mechanical ventilation directly or indirectly.

It was found that some patients showed worse lung function after allogeneic blood transfusion, probably due to the impaired erythrocyte deformability and increased oxygen affinity of stored red blood cells (RBCs) (27, 28). In the rat model, the transfusion of degenerated blood products led to microcirculation disturbance and then blocked the pulmonary capillary (29). Similarly, the microaggregates from degenerated platelets and fibrin led to pulmonary insufficiency (30). The embolism of pulmonary microcirculation from damaged RBCs and microaggregates may be one of the potential causes of PMV in patients with blood transfusion (31–33).

On the other hand, transfusion-related immunomodulation is the direct cause of postoperative infections and multiple organ failure. The plasma from pRBCs induced neutrophils to produce superoxide, enhanced cytotoxicity, and stimulated pulmonary endothelial cells (34, 35). The soluble human leukocyte antigen (HLA) class I molecules circulating in FFP and the proinflammatory cytokines and chemokines (e.g., IL-1, tumor necrosis factor, IL-6, and IL-8) accumulating in PC resulted in diffuse inflammatory and pulmonary dysfunction postoperatively, which then led to PMV (36–39).

It had been proven that older age (≥70 years) means a higher postoperative PMV risk in patients undergoing coronary artery bypass grafting or elective coronary artery surgery, and older age is also an independent risk factor for PMV after total cavopulmanory connection surgery (16, 40, 41). A similar conclusion was found in another medical ICU, where patients with MV extubation failure were older than those with successful extubation (74.0 vs.70.0 years; *p* = 0.003) (42). When assessing PMV risk factors after corrective surgery for tetralogy of Fallot, the higher risk group was found to be younger (12 months, interquartile range 8–19 months) (17). After surgical repair of congenital heart disease, the younger ones [11.95 months (Min–Max range 0.3–158.7 months)] also had a higher risk (43). Age is an important consideration when defining pediatric PMV because it relates to lung maturity (44). The opposite among older persons may be due to a gradual decline in lung function and the body's capacity to stress. We have not yet found whether age can predict postoperative PMV of ATAAD. In all 384 cases, age followed a normal distribution (47.64 ± 10.69 years), with a median age of 47 years (20–80 years). This study confirmed that age was a significant risk factor for PMV of ATAAD after TAR in all three endpoints (PMV ≥24 h, ≥48 h, and ≥72 h). The mean age of patients with PMV ≥24 h was 49.86 ± 11.123 h, and the mean age was higher in PMV ≥48 h or ≥72 h. The likely cause is a gradual decline in pulmonary function and a tolerance decrease to the lengthy, major traumatic procedure.

Only two studies have confirmed that leukocytosis is a risk factor for PMV after cardiovascular surgery, one had PMV ≥24 h after cardiac surgery with CPB, and the other had PMV ≥72 h after acute DeBakey type I aortic dissection surgery (45, 46). Similar results were obtained; preoperative leukocytosis was an independent risk factor for two endpoints of PMV ≥24 h and ≥72 h in ATAAD after TAR. There is no reference research for why there is no predictive value in PMV ≥48 h. Our subsequent studies will include neutrophil count and proportion, C-reactive protein, and other indicators to find the cause.

Many factors lead to PMV, and our research provided a new idea for ATAAD after TAR to predict PMV. However, for most cardiovascular surgeries requiring blood products transfusion, reducing or restricting the transfusion volume without reference is not a reasonable way to reduce PMV incidence. Restrictive pRBC transfusion failed to show advantages and increased the risk of mortality sometimes (47–49). It may be a more direct way to avoid using the long-stored red cells in a long and traumatic surgery (50). We also considered removing excessive hemolysis products and anticoagulants by the purification system before transfusion to reduce post-transfusion complications (51, 52). More targeted research is needed to facilitate the development of guidelines.

## Conclusions

In patients with ATAAD after TAR, PMV incidence was very high. Blood product transfusion was closely related to postoperative mechanical ventilation time. pRBC and PC transfusions and age increased the incidence of PMV at all three endpoints. FFC had no noticeable impact on PMV.

## Limitations

This was a single-center study, which is the main limitation of this research. As a retrospective study, there was a lack of data on the transfusion time (intraoperative or postoperative) and how long the blood products were stored, especially pRBCs.

## Data Availability Statement

The original contributions presented in the study are included in the article/Supplementary Material, further inquiries can be directed to the corresponding author/s.

## Ethics Statement

The studies involving human participants were reviewed and approved by Medical Ethical Committee of the Beijing Anzhen Hospital of Capital Medical University (2020100X). Written informed consent for participation was not required for this study in accordance with the national legislation and the institutional requirements.

## Author Contributions

CLi, YG, LS, and JZhu: conception and design. CLi, YG, LS, YL, JZhe, and JZhu: provision of research materials or patients. QX, YZ, CLu, and RG: data collection and analysis. All authors: manuscript writing. All authors contributed to the article and approved the submitted version.

## Funding

This work was supported by the Beijing Major Science and Technology Projects from the Beijing Municipal Science and Technology Commission (No. Z191100006619093) and the Natural Science Foundation of China (No. 81970393).

## Conflict of Interest

The authors declare that the research was conducted in the absence of any commercial or financial relationships that could be construed as a potential conflict of interest.

## Publisher's Note

All claims expressed in this article are solely those of the authors and do not necessarily represent those of their affiliated organizations, or those of the publisher, the editors and the reviewers. Any product that may be evaluated in this article, or claim that may be made by its manufacturer, is not guaranteed or endorsed by the publisher.
