# Supplementary material for: Blood Transfusion Predicts Prolonged Mechanical Ventilation in Acute Stanford Type A Aortic Dissection Undergoing Total Aortic Arch Replacement
Source: Front Cardiovasc Med. 2022 Apr 15;9:832396. doi: 10.3389/fcvm.2022.832396 (PMC9053570; doi:10.3389/fcvm.2022.832396)
Supplement: Supplementary file 1 [file Table_1.DOCX]

Supplementary Table 1 The differences in blood products transfusion volume in main complications.

| **Main complications** | **pRBCs (units)** | **P-value** | **FFP (mL)** | **P-value** | **PC (units)** | **P-value** |
| --- | --- | --- | --- | --- | --- | --- |
| **AKI** |  | **<0.001** |  | **0.006** |  | **<0.001** |
| No (n=181, 47.10%) | 4.00 (2.00-9.00) |  | 400.00 (0-800.00) |  | 0 (0-1.00) |  |
| Yes (n=203, 52.90%) | 10.00 (4.00-20.00) |  | 400.00 (200.00-1000.00) |  | 1.00 (0.00-3.00) |  |
| **KIDGO3** |  | **<0.001** |  | **0.004** |  | **<0.001** |
| No (n=307, 79.90%) | 6.00 (4.00-10.00) |  | 400.00 (0-800.00) |  | 0 (0-1.00) |  |
| Yes (n=77, 20.10 %) | 16.00 (10.00-22.00) |  | 600.00 (400.00-1000.00) |  | 2.00 (1.00-4.00) |  |
| **CRRT** |  | **<0.001** |  | **0.001** |  | **<0.001** |
| No (n=323, 84.10%) | 6.00 (4.00-12.00) |  | 400.00 (0-800.00) |  | 0 (0-1.00) |  |
| Yes (n=61,15.90 %) | 18.00 (11.00-23.50) |  | 600.00 (400.00-1200.00) |  | 2.00 (1.00-5.00) |  |
| **Secondary thoracotomy** |  | **<0.001** |  | **<0.001** |  | **<0.001** |
| No (n=346, 90.10%) | 6.00 (4.000-12.00) |  | 400.00 (0-800.00) |  | 0.00 (0-1.00) |  |
| Yes (n=38, 9.90%) | 20.00 (14.00-28.50) |  | 1200.00 (600.00-1700.00) |  | 2.00 (1.00-4.00) |  |
| **Stroke** |  | **<0.001** |  | **0.042** |  | **0.001** |
| No (n=344, 89.60%) | 6.50 (4.00-12.00) |  | 400.00 (0-800.00) |  | 0 (0-1.00) |  |
| Yes (n=40, 10.40%) | 14.00 (8.00-22.00) |  | 600.00 (400.00-1200.00) |  | 2.00 (0-3.00) |  |
| **Paraplegia and paraparesis** |  | 0.269 |  | **0.003** |  | **<0.001** |
| No (n=359, 93.50%) | 8.00 (4.00-14.00) |  | 400.00 (0-800.00) |  | 0 (0-2.00) |  |
| Yes (n=25, 6.50%) | 18.00 (6.00-25.50) |  | 600.00 (100.00-1200.00) |  | 2.00 (1.00-4.00) |  |
| **Mortality** |  | **<0.001** |  | **<0.001** |  | **0.002** |
| No (n=355, 92.40%) | 7.00 (4.00-12.00) |  | 400.00 (0-800.00) |  | 0 (0-2.00) |  |
| Yes (n=29, 7.60%) | 16.00 (10.00-24.50) |  | 1000.00 (500.00-1600.00) |  | 2.00 (0-3.00) |  |

AKI, acute kidney injury; KDIGO, Kidney Disease: Improving Global Outcomes; CRRT, continuous renal replacement therapy, pRBCs, packed red blood cells; FFP, fresh frozen plasma; PC, platelet concentrate.

Data were present as n (%), or median (interquartile range) according to variable category. Differences were considered statistically significant for P-values of <0.05, which are shown in bold.
